# Preoperative endogenous testosterone density predicts disease progression from localized impalpable prostate cancer presenting with PSA levels elevated up to 10 ng/mL

**DOI:** 10.1007/s11255-022-03366-3

**Published:** 2022-10-05

**Authors:** Antonio Benito Porcaro, Alberto Bianchi, Giovanni Mazzucato, Sebastian Gallina, Emanuele Serafin, Alessandro Tafuri, Clara Cerrato, Andrea Panunzio, Stefano Vidiri, Damiano D’Aietti, Rossella Orlando, Davide Brusa, Matteo Brunelli, Salvatore Siracusano, Maria Angela Cerruto, Alessandro Antonelli

**Affiliations:** 1 Department of Urology, University of Verona, Azienda Ospedaliera Universitaria Integrata, Piazzale Stefani 1, 37126 Verona, Italy; 2 Department of Urology, Vito Fazzi Hospital, 73110 Lecce, Italy; 3 Department of Pathology, University of Verona, Azienda Ospedaliera Universitaria Integrata, Verona, Italy; 4 Department of Life, Health and Environmental Sciences, University of L’Aquila, L’Aquila, Italy

**Keywords:** Prostate cancer, Radical prostatectomy, Endogenous testosterone, Prostate volume, Endogenous testosterone density, Prostate cancer progression

## Abstract

**Objective:**

To investigate endogenous testosterone density (ETD) predicting disease progression from clinically localized impalpable prostate cancer (PCa) presenting with prostate-specific antigen (PSA) levels elevated up to 10 ng/mL and treated with radical prostatectomy.

**Materials and methods:**

In a period ranging from November 2014 to December 2019, 805 consecutive PCa patients who were not under androgen blockade had endogenous testosterone (ET, ng/dL) measured before surgery. ETD was evaluated as the ratio of ET on prostate volume (PV). Unfavorable disease was defined as including ISUP ≥ 3 and/or seminal vesicle invasion in the surgical specimen. The risk of disease progression was evaluated by statistical methods.

**Results:**

Overall, the study selected 433 patients, of whom 353 (81.5%) had available follow-up. Unfavorable disease occurred in 46.7% of cases and was predicted by tumor quantitation features that were positively associated with ETD. Disease progression, which occurred for 46 (13%) cases, was independently predicted only by ETD (hazard ratio, HR = 1.037; 95% CI 1.004–1.072; *p* = 0.030) after adjusting for unfavorable disease. According to a multivariate model, ETD above the third quartile was confirmed to be an independent predictor for PCa progression (HR = 2.479; 95% CI 1.355–4.534; *p* = 0.003) after adjusting for unfavorable disease. The same ETD measurements, ET mean levels were significantly lower in progressing cancers.

**Conclusions:**

In this particular subset of patients, increased ETD with low ET levels, indicating androgen independence, resulted in a more aggressive disease with poorer prognosis.

**Supplementary Information:**

The online version contains supplementary material available at 10.1007/s11255-022-03366-3.

## Introduction

Prostate cancer (PCa) is a major health problem among aged males resulting in being the second most diagnosed tumor and is graded according to the International Society of Urological Pathology (ISUP) [1, 2]. After diagnosis, patients are stratified into prognostic categories according to the European Association of Urology (EAU) and the National Comprehensive Cancer Network (NCCN) guidelines, which are not equivalent [1, 2]. Risk categories are computed according to routinely evaluated parameters including prostate-specific antigen (PSA), ISUP grade group, and TNM clinical staging system [1, 2]. Treatment options may vary from monitoring strategies [active surveillance (AS) and watchful waiting] up to active treatments including radical prostatectomy (RP) eventually associated with extended pelvic lymph node dissection (ePLND), radiation therapy (RT), or brachytherapy [1, 2]. Active treatments may trigger treatment-related dissatisfaction among the patients, as has been recently shown by a large prospective study [3]. The use of molecular tumor analysis is still controversial in patients with unfavorable prognosis factors. The latter includes tumor upgrading and upstaging in the surgical specimen [1, 2]. Impalpable disease with PSA elevated up to 10 ng/mL, which occurs frequently among all risk groups with differences related only to the ISUP system, represents a challenging treatment subset of patients for the urologist and radiation oncologist [1–3].

The risk of PCa has been associated with genetic, dietary, environmental, metabolic, and hormonal factors, with endogenous testosterone (ET) being the main involved androgen [1, 2, 4]. The relation of ET and unfavorable disease in the surgical specimen has been investigated by our group showing positive or inverse association with the risk according to the factor being evaluated as a continuous or categorical variable [5–7]. Recently, we have focused our research on the association between ET density (ETD), defined as the ratio of ET on prostate volume (PV), and tumor quantitation features showing positive associations along all risk groups [8–12]. The present study investigate the potential prognostic role of ETD in predicting disease progression from clinically localized not palpable PCa with PSA levels elevated up to 10 ng/mL and surgically treated.

## Materials and methods

### Patient population, data collection, and evaluation of parameters

The study was approved by institutional review board. Informed consent was obtained by all subjects. Data were collected prospectively, but evaluated retrospectively. In a period ranging from November 2014 to December 2019, 805 consecutive PCa patients who were not under androgen blockade had ET (ng/dL) measured at our laboratory before surgery. The test was performed at least 1 month after biopsies between 8.00 and 8.30 a.m. by radioimmunoassay. PSA (ng/mL), age (years), body mass index (BMI; kg/m^2^), PV (mL) and percentage of biopsy-positive cores (BPC), and the percentage ratio of positive and total taken cores (%) were evaluated for each case. PV was calculated by transrectal ultrasound (TRUS) standard methods. Biopsies performed elsewhere were assessed for the number of cores, tumor grade, and PV, which was measured by the transrectal approach. The 14-core transperineal biopsy technique was used. In each case, the ratios of BPC, PSA, and ET with PV were calculated and relative densities were indicated as BPCD (%/mL), PSAD (ng/ml^2^), and ETD [ng/(dL × mL)]. Clinical staging was assessed by the 2017 version of the TNM system with clinical T stage only referring to digital rectal examination findings. Patients were classified into risk classes as recommended by EAU guidelines [1].

Preoperative physical status was evaluated by the American Society of Anesthesiologists (ASA) system [13]. Surgery, which was delivered by robot-assisted (RARP) or open approach, was performed by experienced surgeons. Extended pelvic lymph dissection (ePLND) was performed according to guidelines [1, 2]. Lymph nodes were removed and submitted in separate packages according to standard anatomical template (including external iliac, internal iliac and obturator, Marcille’s common iliac, and Cloquet’s nodal stations, bilaterally) [5–7]. Specimens including prostate and dissected lymph nodes were placed in formalin and evaluated by a dedicated pathologist. Prostates were weighted and tumors were graded according to the ISUP system [1, 2]. Tumor quantitation was assessed as tumor load (TL), defined as the percentage of prostate affected by cancer; specifically, our dedicated pathologist assessed tumor quantitation by visual estimation of the glass slides after all microscopically identifiable foci of carcinoma were circled with a marked pen, as considered by ISUP association [14]. Tumor load density (TLD) was calculated as the ratio of TL on prostate weight (%/g). Surgical margins were considered positive when cancer invaded the inked surface of the specimen. Removed lymph nodes were counted and assessed for cancer invasion.

### Study design

The study aimed to test the hypothesis of ETD as a prognostic factor for PCa progression in patients with impalpable organ-confined disease and with PSA elevated up to 10 ng/mL. Overall, 433 patients met the study criteria. In the surgical specimen, unfavorable disease was defined as including ISUP grade group ≥ 3 and/or seminal vesicle invasion (SVI). Patients were followed up, according to EAU recommendations [1]. Specifically, clinical history and PSA measurements were obtained at 3, 6, and 12 months after treatment, then every 6 months for 3 years and yearly thereafter. At PSA persistence/recurrence, imaging modalities were considered to restage the disease and plan further treatments. Disease progression was defined as any event leading to recurrence and included biochemical recurrence/persistence and/or local recurrence and/or distant metastases. According to EAU guidelines, biochemical recurrence after surgery was defined as PSA ≥ 0.2 ng/mL with a second confirmatory level of PSA > 0.2 ng/mL [1].

### Statistical analysis

Continuous variables were measured for medians and interquartile ranges (IQR). Categorical factors were assessed for frequencies (percentages). Associations with continuous and categorical variables were evaluated according to tests of Mann–Whitney and Chi-squared of Pearson, respectively. The length of time between surgery and the clinical outcome of interest (disease progression) or the last follow-up was measured as time to event occurrence. Univariate and multivariate Cox proportional hazards models were used to estimate the association of clinical and pathological factors with the risk of disease progression; hazards ratios and relative 95% confidence intervals (CI) were evaluated. The software used to run the analysis was IBM-SPSS version 26. All tests were two sided with *p* < 0.05 considered to indicate statistical significance.

## Results

### Demographics of the patient population stratified by unfavorable disease in the surgical specimen

The demographics of patients stratified by unfavorable disease are described in Table 1. The selected population had a median age of 65 years with a median PSA of 5.9 ng/mL and a median BPC of 28%. A biopsy ISUP grade group ≥ 3 was detected in 106 cases (24.5%). According to the EAU system, 172 patients (39.7%) were considered to be at low risk, 234 (54%) at intermediate risk, and 27 (6.2%) at high risk. Median PV, PSAD, BPCD, and ETD were 40 mL, 0.14 ng/(mL × mL), 0.70 (%/mL), and 9.8 [ng/(dL × mL)], respectively. According to the ASA system, patients were classified as grade 1, 2, and 3 in 45 (10.4%), 350 (80.8%), and 38 (8.8%) cases. Surgery was delivered by RARP in 386 (89.1%) cases. In the surgical specimen, ISUP grade group ≥ 3, seminal vesicle invasion (SVI), and positive surgical margins were detected in 197 (45.5%), 34 (7.9%), and 104 (24%) patients. Of 280 patients staged anatomically, 18 (6.4%) had lymph node invasion. Median TL and TLD were 15% and 0.27%/g.

**Table 1:** Patient population including operated impalpable prostate cancer associated with PSA levels elevated up to 10 ng/mL stratified by unfavorable disease including ISUP ≥ 3 and/or seminal vesicle invasion

Factors (*)	Population	Favorable disease	Unfavorable disease	*p* value
Number	433	231 (53.3)	202 (46.7)	
Clinical features				
Endogenous testosterone; ET (ng/dL)	403 (313.5–520.5	387.6 (308.3–522)	414.6 (319.5–521.3)	0.421
ET density; ETD (ng/(dL × mL))	9.8 (6.7–14.1)	9.8 (6.6–13.7)	9.8 (6.8–15.2)	0.286
Age (years)	65 (60–70)	64 (59–69)	67 (61.7–71)	**0.001**
Body mass index; BMI (kg/m^2^)	25.8 (23.9–28.1)	25.9 (24.1–27.7)	25.7 (23.8–28.4)	0.606
Prostate volume; PV (mL)	40 (30–52)	40 (31–54)	40 (29–50)	0.151
Prostate-specific antigen; PSA (ng/mL)	5.9 (4.6–7.4)	5.8 (4.6–7.3)	6.0 (4.6–7.7)	0.383
PSA density; PSAD (ng/(mL × mL))	0.14 (0.10–0.19)	0.13 (0.10–0.18)	0.15 (0.11–0.20)	0.072
Percentage of biopsy positive cores; BPC (%)	28 (17–43)	25 (14–12)	29 (20–47.7)	**0.012**
BPC density; BPCD (%/mL)	0.70 (0.37–1.21)	0.60 (0.33–1.16)	0.74 (0.45–1.28)	**0.012**
ISUP grade group				** < 0.0001**
ISUP ≤ 2	327 (75.5)	210 (90.9)	117 (57.9)	
ISUP ≥ 3	106 (24.5)	21 (9.1)	85 (42.1)	
EAU risk classes				** < 0.0001**
Low	172 (39.7)	121 (52.4)	51 (25.2)	
Intermediate	234 (54.0)	105 (45.5)	129 (63.9)	
High	27 (6.2)	5 (2.2)	22 (10.9)	
Prostate pathological features				
Prostate weight; PW (grams; gr)	53 (41–66)	54 (40–67)	53 (42–65)	0.929
Tumor load; TL (%)	15 (10–25)	10 (8–20)	20 (10–30)	** < 0.0001**
Tumor load density; TLD (%/gr)	0.27 (0.15–0.51)	0.22 (0.12–0.40)	0.36 (0.19–0.55)	** < 0.0001**
ISUP grade group				** < 0.0001**
ISUP ≤ 2	236 (54.5)	231 (100)	5 (2.5)	
ISUP ≥ 3	197 (45.5)		197 (97.5)	
Pathological stage (pT)				** < 0.0001**
pT2	366 (84.5)	225 (97.4)	141 (69.8)	
pT3a	33 (7.6)	6 (2.6)	27 (13.4)	
pT3b	34 (7.9)		34 (16.8)	
Surgical margins; R				**0.018**
Negative (R0)	329 (76)	186 (80.5)	143 (70.8)	
Positive (R1)	104 (24)	45 (19.5)	59 (29.2)	

Statistically significant results are shown in bold

*ISUP* International Society of Urologic Pathology tumor grade group formulation, *EAU* European Association of Urology

(*), continuous variables are reported as medians (IQR, interquartile ranges) and categorical factors as frequency (percentage)

### Factors associated with unfavorable disease and tumor quantitation density features

Overall, unfavorable disease occurred in 202 cases (46.7%) and it was associated with age and features related to tumor grade (ISUP grade group ≥ 3 at either biopsy and pathology), tumor quantitation (BPC, BPCD, TLD), and positive surgical margins (R1). However, on multivariate analysis, the risk of unfavorable disease was only predicted by age (odds ratio, OR = 1.048; 95% CI 1.013–1.083; *p* = 0.006), biopsy ISUP grade group ≥ 3 (OR = 6.852; 95% CI 4.006–11.718; *p* < 0.0001) and TLD (OR = 2.630; 95% CI 1.356–5.101; *p* = 0.004), as shown in supplementary Table S1. On univariate analysis, TLD positively correlated with ETD (Pearson’s correlation coefficient, *r* = 0.263; *p* < 0.0001), PSAD (*r* = 0.271; *p* < 0.0001), pathology ISUP ≥ 3 (*r* = 0.130; *p* = 0.007), pT (*r* = 0.162; *p* = 0.001) and R1 (*r* = 0.220; *p* < 0.0001), but not with other factors. As shown in supplementary Table S2, ETD was a positive independent predictor for tumor quantitation density features after adjusting for other factors.

Instead, patients with pT3a cancer were not considered in the unfavorable disease group, because it was not an independent predictor as shown in Tables 1 and 2.

**Table 2:** Prognostic factors associated with the risk of disease progression in operated prostate cancer patients presenting with impalpable disease and PSA elevated up to 10 ng/mL

	No disease progression	Disease progression	Univariate analysis (*)		Multivariate analysis (**)	
Statistics	*N* = 307 (87%)	*N* = 46 (13%)	HR (95% CI)	*P* value	HR (95% CI)	*p* value
ETD	**9.8 (6.8–13.9)**	**11.9 (6.7–17.5)**	**1.033 (1.003–1.064)**	**0.031**	**1.037 (1.004–1.071)**	**0.030**
Age	66 (60–70)	66 (62–70)	1.026 (0.978–1.077)	0.300		
BMI	25.8 (24–28.4)	25.4 (24.2–27.2)	0.932 (0.850–1.022)	0.932		
PV	40 (30–52)	39 (29.5–53)	0.992 (0.974–1.011)	0.433		
PSA	5.9 (4.5–7.3)	6.1 (4.9–7.8)	1.056 (0.903–1.236)	0.495		
PSAD	0.13 (0.10–0.19)	0.15 (0.11–0.21)	10.178 (0.549–188.654)	0.119		
BPC	27 (17–43.7)	29 (17–57)	1.007 (0.994–1.020)	0.301		
BPCD	0.66 (0.36–1.21)	0.77 (0.39–1.84)	1.465 (1.066–2.015)	**0.019**	Not significant (removed)	
ISUP ≤ 2	242 (78.9)	33 (71.7)	Ref		Ref	
ISUP ≥ 3	65 (21.1)	13 (28.3)	1.267 (0.666–2.410)	0.472		
PW	53 (41–65)	53.5 (40–71)	0.996 (0.981–1.012)	0.649		
TLD	0.28 (0.16–0.51)	0.31 (0.15–0.51)	1.688 (0.883–3.150)	0.115		
ISUP ≤ 2	186 (60.6)	14 (30.4)	Ref		Ref	
ISUP ≥ 3	**121 (39.4)**	**32 (69.7)**	**3.488 (1.859–6.546)**	** < 0.0001**	**3.250 (1.665–6.374)**	**0.001**
pT2	270 (87.9)	32 (69.6)	Ref			
pT3a	23 (7.5)	3 (6.5)	1.127 (0.345–3.689)	0.843		
pT3b	**14 (4.6)**	**11 (23.9)**	**4.002 (2.006–7.985)**	** < 0.0001**	**2.208 (1.070–4.558)**	**0.032**
R0	245 (79.8)	28 (60.9)	Ref		Ref	
R1	62 (20.2)	18 (39.1)	2.142 (1.179–3.889)	**0.012**	Nor significant (removed)	

Statistically significant results are shown in bold

See also Table 1

*HR* hazard ratio, *CI* confidence interval

(*), by Cox's proportional hazards; (**), by Cox's proportional hazards through the forward stepwise method

### Endogenous testosterone density as an independent prognostic factor for disease progression

Median (IQR) follow-up, available for 353 (81.5%) patients, was 42 (23–57) months with no significant difference among groups with or without progression. None of the five deaths was related to PCa; 98.6% of patients were alive at censoring time. As illustrated in Table 2, disease progression, which occurred in 46 (13%) cases, was positively associated with ETD, BPCD, pathology ISUP grade group ≥ 3, SVI, and R1 status. However, on multivariate analysis, disease progression was independently predicted only by ETD (hazard ratio, HR = 1.037; 95% CI 1.004–1.072; *p* = 0.030), pathology ISUP ≥ 3 (HR = 3.250; 95% CI 1.665–6.347; *p* = 0.001) and SVI (HR = 2.208; 95% CI 1.070–4.558; 0.032). According to the Cox’s proportional model, we stratified ETD by quartiles and found out that disease progression occurred for levels above the third quartile (HR = 2.780; 95% CI 1.293–5.975; *p* = 0.009), but not for the second (HR = 1.058; 95% CI 0.433–2.587; *p* = 0.901) or third one (HR = 0.963; 95% CI 0.372–2.494; *p* = 0.934), having the first quartile as reference.

**Fig. 1:**
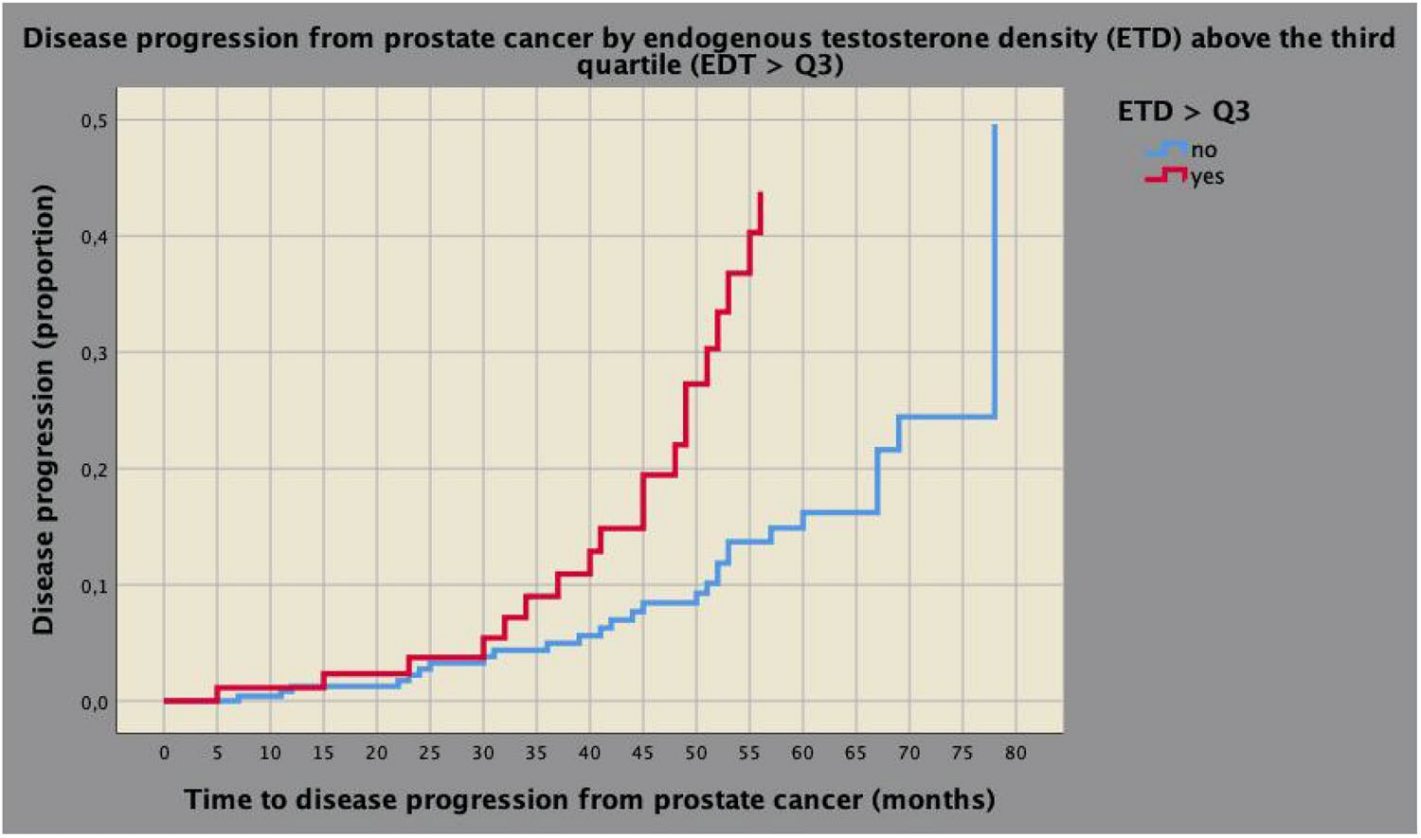
Cumulative risk curves of disease progression from prostate cancer (PCa) stratified by clinical levels of endogenous testosterone density (EDT), for patients presenting with clinically localized not palpable PCa with prostate-specific levels (PSA) elevated up to 10 ng/mL. Patients presenting with ETD levels above the third quartile (see Table 1) showed an increased risk of disease progression. Notably, at a follow-up of 55 months, the risk of disease progression was 40% for patients with EDT above the third quartile, but only 10% for cases presenting with ETD up to the third quartile, as well

Table 3 reports a multivariate model including prognostic factors associated with the risk of disease progression in patients presenting with impalpable clinically localized PCa with PSA levels elevated up to 10 ng/mL. In the model, unfavorable disease was coded at three levels as being absent (level 0), including ISUP ≥ 3 and/or SVI (level 1) or both (level 2). After adjusting for level 1 (HR = 3.350; 95% CI 1.677–6.692; *p* = 0.001) and 2 (HR = 6.793; 95% CI 2.844–16.229; *p* < 0.0001) of unfavorable disease, ETD above the third quartile was confirmed to be an independent prognostic factor for PCa progression (HR = 2.479; 95% CI 1.355–4.534; *p* = 0.003). Further details are explained in the referred table. The cumulative risk curves of time to disease progression for PCa stratified by different levels of unfavorable disease and by ETD above the third quartile are illustrated in Fig. 1 and Supplementary Fig. 1. The inverse linear relation between ET and ETD in patients with PCa progression compared to those without is illustrated in Supplementary Fig. 2; mean ET levels were significantly lower for the former compared with the latter, although showing the same ETD levels.

**Table 3:** Prognostic factors predicting disease progression in patients with impalpable disease with PSA elevated up to 10 ng/mL

	No disease progression	Disease progression	Multivariate analysis	
Statistics	*N* (%)	*N* (%)	HR (95% CI)	*p *value
ETD up to the third quartile	234 (89.7)	27 (10.3)	1	
ETD above the third quartile	73 (79.3)	19 (20.7)	2.479 (1.355–4.534)	0.003
Unfavorable disease absent	183 (93.8)	12 (6.2)	1	
ISUP > 2 and/or SVI	113 (81.9)	25 (18.1)	3.350 (1.677–6.692)	0.001
ISUP > 2 and SVI	11 (55)	9 (45)	6.793 (2.844–16.229)	< 0.0001

See also Table 1

*HR* hazard ratio, *CI* confidence interval

## Discussion

The association of ET with aggressive PCa is controversial, as controlled studies are missing. Specifically, the controversy is supported by results demonstrating that the association may be inverse, positive, or null with the former being the most frequent occurrence [15, 16]. Several studies have shown inverse associations of ET with features of unfavorable disease in the surgical specimen such as tumor upgrading and upstaging; however, these investigations were limited by the retrospective nature and heterogeneity of the cohorts that were historical with a small number of cases and/or for not accounting for diurnal variations of ET [15, 16]. In biopsy and surgical specimens, tumor quantitation is an important parameter for evaluating unfavorable PCa; however, only the former is extensively used, while the latter, although reported routinely, is rarely considered [1, 2, 14]. Biopsy tumor quantitation is included in the NCCN and CAPRA system as well as in nomograms for predicting lymph node invasion [17, 18]. Nevertheless, a recent multicenter study showed that currently available nomograms performed worse than old ones, because the predictive power was not increased by mpMRI, which showed to be highly operator dependent [19]. Biopsy tumor quantitation features may impact stratifying PCa risk categories [20]. We have already shown that ETD is associated with unfavorable disease by the positive correlation with tumor density quantitation factors including BPCD and TLD; we have also demonstrated that unfavorable disease is associated with lower ET levels, independently by ETD measurements [8–12] and the present study has confirmed these results.

Unfavorable disease was most frequently detected in the intermediate EAU risk class (63.9%), followed by the low risk one (25.2%), and finally by the high-risk group (10.9%). Tumor density features were the main factors associated with unfavorable disease, together with biopsy ISUP grade group ≥ 3. Nevertheless, although biopsy ISUP grade group ≥ 3 was the main predictor of unfavorable disease, 57.9% of cases classified as ISUP grade group ≤ 2 had tumor upgrading eventually associated with seminal vesicle invasion in the surgical specimen, thus suggesting that biopsy ISUP grade group and clinical staging are still an issue when dealing with such a set of patients. Our study showed that ETD together with PSAD was the only clinical factor associated with cancer density features, including TLD and BPCD. The risk of detecting high tumor load increased when ETD and PSAD were higher. As a result, they both indirectly predicted unfavorable disease in the surgical specimen.

Although actual tumor grade formulation predicts PCa natural history at diagnosis, tumor upgrading is still an issue for drawbacks on disease progression. As tumor grade increases through the ISUP system, the probability of disease progression increases; the 5-year biochemical risk-free survival of grade groups 1–5 after RP was 96%, 88%, 63%, 48%, and 26% [1, 2]. Although a large study of 1113 patients has shown that tumor upgrading is associated with adverse pathological features and biochemical progression, it suffered limitations: historical and non-homogenous cohorts were included, PV and PSAD were not measured, and the study was retrospective [21]. Associations between preoperative ET levels and risk of PCa progression are even more controversial [15, 16]. In particular, the association might be absent or present with the latter showing an inverse or a positive prediction for the factor evaluated as a categorical or continuous variable; however, these studies were all severely biased by a limited number of cases, heterogeneity of outdated cohorts, the retrospective nature, and not evaluating tumor density factors [22–28]. This study investigated PCa progression by routine factors also including ETD in a highly selected cohort of clinically localized impalpable disease with PSA elevated up to 10 ng/mL. This subset of patients was mostly represented by the intermediate EAU risk class (54%), followed by the low-risk (39.7%) and finally by the high-risk group (6.2%) of patients. Notably, ETD was the only clinical factor predicting the risk of disease progression after adjusting for unfavorable disease including ISUP ≥ 3 and seminal vesicle invasion. Interestingly, unfavorable disease coded at three levels showed prognostic prediction on PCa progression. As shown in Table 3, the risk of disease progression increased from 6.2% (level 0) through 18.1% (level 1) up to 45.0% (level 2). As the level of unfavorable disease increased, the risk of PCa progression also increased. Nevertheless, the risk of PCa progression also increased through ETD levels such that patients presenting with levels above the third quartile showed a 20.7% risk of cancer progression compared with cases without (10.2%) after adjusting for levels of unfavorable disease. ET levels were significantly lower for patients who had a disease progression compared with others without progression, although ETD was the same for both groups. This is the first study demonstrating associations between preoperative ETD and PCa progression, but confirmatory trials are required.

Our findings may explain basic science theories of PCa biology. ETD is associated with high TLD; moreover, patients having the same ETD experienced higher TLD and disease progression for low ET levels. These results support theories that underpin the pivotal role of low ET levels on prostate androgen-dependent cells. A decrease in ET levels occurs physiologically in middle-aged males [29]. Several studies investigating the associations between ET and aggressive PCa have shown that prostate growth is strictly dependent on ET at very low levels, but when ET levels decrease down to critical points, it has drawbacks on differentiation and division of androgen-dependent cells; furthermore, as far as prostate cells are continuously exposed to low ET levels, the risk of cancer induction and progression increases [30].

The results of our study have clinical implications. The cohort is representative of a very favorable subset of patients running from the low through the intermediate up to the high-risk classes, which occur frequently in daily practice and implicate treatment decisions for urologists and radiation oncologists. Patients presenting with ETD levels above the third quartile with low ET levels are at increased risk of disease progression along all risk classes of the two main systems of NCCN and EAU. These results might be helpful for clinicians to decide on the appropriate management of patients at diagnosis as well as after primary treatments. These results may be integrated into mpMRI findings, but controlled studies are required.

Our study has several limitations. Prostate volumes were not all measured at our institution. ET was measured only once and not on a periodic base. A central pathology review of external biopsies was not performed. Additionally, mpMRI was not available for all patients and genomic tests were not performed. The percentage of pattern 4 in biopsy ISUP grade group 2 was not evaluated. Analysis of maximal cancer involvement of each core, which is an important feature for assessing indolent cancers, was not performed for not being available in all patients [20]. Finally, the retrospective nature of the study. Our study has several strengths as well. All prostate specimens were assessed by our dedicated pathologist. ET was measured in the morning, the appropriate time for evaluating the levels of the hormone, which decreases in the afternoon. Data were prospectively collected. The study was single center including Caucasian patients with ET measurements being performed at our laboratory.

## Conclusions

In PCa patients presenting with PSA levels elevated up to 10 ng/mL but with impalpable cancer, ETD was an independent predictor of disease progression. The risk of disease progression increased as ETD increased, but ET levels were significantly lower for progressing cancers. In this subset of patients, increased ETD and low ET levels, indicating androgen independence, resulted in a more aggressive disease with poorer prognosis.

## Supplementary Information

Below is the link to the electronic supplementary material.

Supplementary file1 Supplementary Fig. 1 Cumulative risk curves of disease progression from prostate cancer stratified by levels of unfavorable disease in the surgical specimen in patients presenting with not palpable clinically localized disease and prostate-specific antigen levels (PSA) elevated up to 10 ng/mL. Levels of unfavorable disease were coded as absent, including ISUP grade group ≥ 3 and/or seminal vesicle invasion as well as either ISUP ≥ 3 and seminal vesicle invasion, as shown in Table 3. The risk of disease progression increased as levels of unfavorable disease increased, accordingly. Notably, at a follow-up of 60 months, disease progression was above 50% for ISUP ≥ 3 with seminal vesicle invasion, 30% for ISUP ≥ 3 and/or seminal vesicle invasion, and only 11% for unfavorable disease being absent. Supplementary Fig. 2 Linear relations between endogenous testosterone density (ETD) and endogenous testosterone stratified by disease progression. Patients with disease progression had significantly lower slope of the regression line (regression coefficient, rc = 8.971; 95% CI: 4.642 – 13.300; p < 0.0001) compared with cases without PCa progression (rc = 14.719; 95% CI: 12.797 – 16.641; p < 0.0001). As a result, mean endogenous testosterone levels were significantly lower for subjects with disease progression compared with patients without, although having the same ETD levels, as well (DOCX 126 KB)

Supplementary file2 (DOCX 22 KB)
