# Supplementary figures and images for: Preoperative endogenous testosterone density predicts disease progression from localized impalpable prostate cancer presenting with PSA levels elevated up to 10 ng/mL
Source: Int Urol Nephrol. 2022 Oct 5;55(1):85–92. doi: 10.1007/s11255-022-03366-3 (PMC9807534; doi:10.1007/s11255-022-03366-3)

**Supplementary Fig. 1**


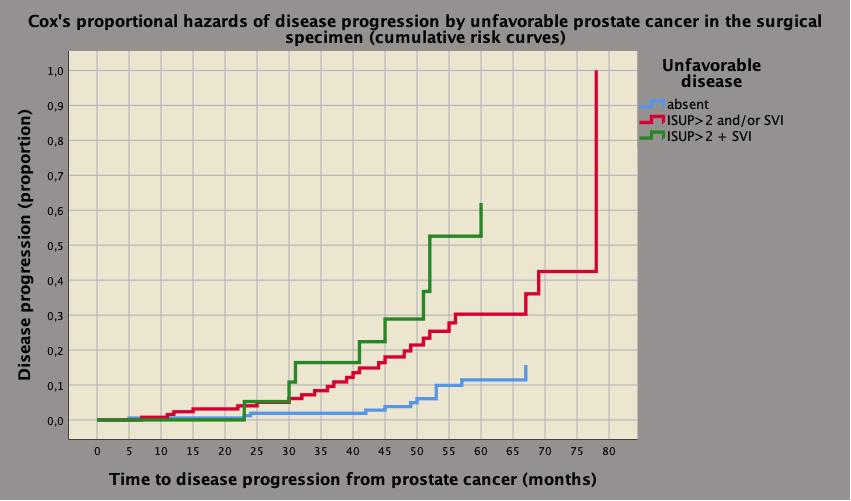


**Supplementary Fig. 2**


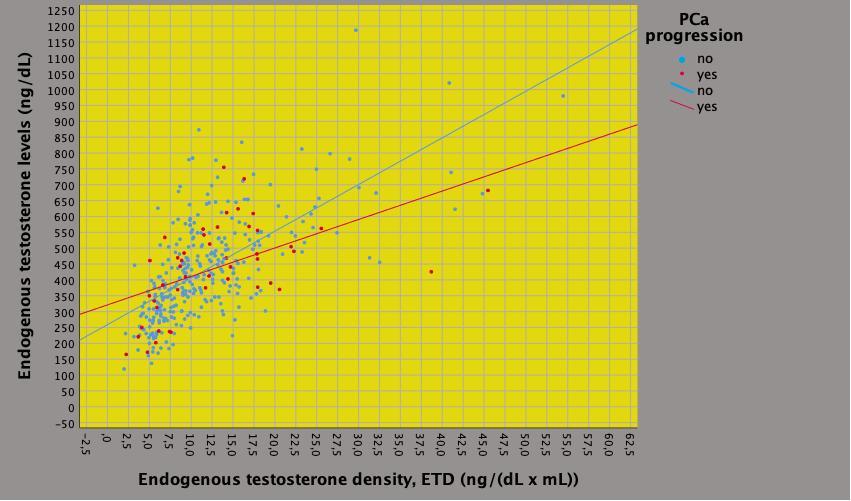

Supplement: Supplementary file 1 — Supplementary file1 Supplementary Fig. 1 Cumulative risk curves of disease progression from prostate cancer stratified by levels of unfavorable disease in the surgical specimen in patients presenting with not palpable clinically localized disease and prostate-specific antigen levels (PSA) elevated up to 10 ng/mL. Levels of unfavorable disease were coded as absent, including ISUP grade group ≥ 3 and/or seminal vesicle invasion as well as either ISUP ≥ 3 and seminal vesicle invasion, as shown in Table 3. The risk of disease progression increased as levels of unfavorable disease increased, accordingly. Notably, at a follow-up of 60 months, disease progression was above 50% for ISUP ≥ 3 with seminal vesicle invasion, 30% for ISUP ≥ 3 and/or seminal vesicle invasion, and only 11% for unfavorable disease being absent. Supplementary Fig. 2 Linear relations between endogenous testosterone density (ETD) and endogenous testosterone stratified by disease progression. Patients with disease progression had significantly lower slope of the regression line (regression coefficient, rc = 8.971; 95% CI: 4.642 – 13.300; p < 0.0001) compared with cases without PCa progression (rc = 14.719; 95% CI: 12.797 – 16.641; p < 0.0001). As a result, mean endogenous testosterone levels were significantly lower for subjects with disease progression compared with patients without, although having the same ETD levels, as well (DOCX 126 KB) [file 11255_2022_3366_MOESM1_ESM.docx]
