# Supplementary material for: Preoperative endogenous testosterone density predicts disease progression from localized impalpable prostate cancer presenting with PSA levels elevated up to 10 ng/mL
Source: Int Urol Nephrol. 2022 Oct 5;55(1):85–92. doi: 10.1007/s11255-022-03366-3 (PMC9807534; doi:10.1007/s11255-022-03366-3)
Supplement: Supplementary file 2 — Supplementary file2 (DOCX 22 KB) [file 11255_2022_3366_MOESM2_ESM.docx]

| **Table S1. Factors predicting prostate cancer unfavorable disease including ISUP** ≥ 3 **and/or seminal vesicle invasion in the surgical specimen of operated patients with impalpable disease and PSA elevated up to 10 ng/mL** | | | | |
| --- | --- | --- | --- | --- |
|  | ***Univariate analysis*** | | ***Multivariate analysis (*)*** | |
|  | OR (95% CI) | *P*-value | OR (95% CI) | *P*-value |
| Age | 1,056 (1,024 - 1,089) | 0,001 | 1,048 (1,103 - 1,083) | 0,006 |
| BPC | 1,012 (1,002 - 1,022) | 0,017 | not significant (removed) |  |
| BPCD | 1,366 (1.037 - 1,799) | 0,027 | not significant (removed) |  |
| ISUP > 2 | 7,265 (4,283 - 12,323) | <0,0001 | 6,852 (4,006 - 11,718) | <0,0001 |
| TLD | 2,388 (1,283 - 4,443) | 0,006 | 2,630 (1,356 - 5,101) | 0,004 |
| R1 | 1,705 (1,093 - 2,661) | 0,019 | not significant (removed) |  |
| Legend: (*), by the Wald forward step method; OR, odds ratio; IC, interval confidence; see also Table 1 | | | | |

| **Table S2. Associations of tumor density factors with endogenous density features and other pathological factors in impalpable prostate cancer with PSA levels elevated up to 10 ng/mL and treated with radical prostatectomy (n = 433)** | | | | | | | | |
| --- | --- | --- | --- | --- | --- | --- | --- | --- |
|  | ***Tumor load density in the surgical specimen*** | | | |  | ***Percentage of biopsy positive cores density*** | | |
|  | Univariate analysis |  | Multivariate analysis (*) |  | Univariate analysis |  | Multivariate analysis (*) | |
| *Statistics* | rc (95% CI) | *p*-value | rc (95% CI) | *p*-value | rc (95% CI) | *p*-value | rc (95% CI) | *p*-value |
| ETD | 0,013 (0,008; 0,017) | <0,0001 | 0,008 (0,003; 0,013) | 0,001 | 0,044 (0,035; 0,052) | <0,0001 | 0,024 (0,016; 0,033) | <0,0001 |
| PSAD | 1,150 (0,763; 1,536) | <0,0001 | 0,712 (0,288; 1,136) | <0,0001 | 3,983 (3,251; 4,714) | <0,0001 | 2,311 (1,548; 3,075) | <0,0001 |
| ISUP > 2 (**) | 0,090 (0,025; 0,155) | 0,007 | 0,068 (0,006; 0,129) | 0,030 |  |  | removed |  |
| pT3a | 0,152 (0,030; 0,273) | 0,015 | removed |  | 0,161 (-0,089; 0',402) | 0,206 | removed |  |
| pT3b | 0,168 (0,048; 0,288) | 0,006 | removed |  | 0,345 (0,098; 0,592) | 0,006 | removed |  |
| R1 | 0,177 (0,103; 0,251) | <0,0001 | 0,139 (0,067; 0,211) | <0,001 | 0,196 (0,041; 0,352) | 0,013 | removed |  |
| TLD |  |  |  |  | 0,855 (0,791; 1,031) | <0,0001 | 0,572 (0,407; 0,737) | <0,0001 |
| Legend: rc, regression coefficients; CI, confidence interval; (*) by the forward stepwise method; see also Table 1 | | | | | | | | |
